# Characterization and chemoproteomic profiling of protein O-GlcNAcylation in SOD1-G93A mouse model

**DOI:** 10.1186/s10020-025-01134-4

**Published:** 2025-02-28

**Authors:** Yi Hao, Zhongzhong Li, Xinyan Du, Qingsong Xie, Dongxiao Li, Shaoyuan Lei, Yansu Guo

**Affiliations:** 1 National Glycoengineering Research Center, Shandong University, Qingdao, Shandong China; 2 Beijing Geriatric Healthcare and Disease Prevention Center, Xuanwu Hospital, Capital Medical University, Changchun Street 45, Beijing, China; 3 Department of Neurology, The First Hospital of Hebei Medical University, Shijiazhuang, Hebei China; 4 Evidence-Based Medicine Center, Xuanwu Hospital, Capital Medical University, Beijing, China; 5 Beijing Municipal Geriatric Medical Research Center, Beijing, China

**Keywords:** O-linked β-*N*-acetylglucosamine (O-GlcNAc), Chemoproteomics, Amyotrophic lateral sclerosis (ALS), SOD1-G93A mice, Chemoenzymatic labeling, Click chemistry

## Abstract

**Background:**

Amyotrophic lateral sclerosis (ALS) is a devastating motor neuron disease. Protein O-linked β-*N*-acetylglucosamine (O-GlcNAc) modification has been found to affect the processing of several important proteins implicated in ALS. However, the overall level and cellular localization of O-GlcNAc during ALS progression are incompletely understood, and large-scale profiling of O-GlcNAcylation sites in this context remains unexplored.

**Methods:**

By using immunostaining analysis and chemoenzymatic labeling-based quantitative chemoproteomics, we assayed O-GlcNAcylation dynamics of lumbar spinal cords from SOD-G93A mice and their non-transgenic (NTG) littermates, the most widely used animal model for studying ALS pathogenesis.

**Results:**

We discovered that the global O-GlcNAcylation was significantly reduced at the disease end stage. Correlatively, a great increase of OGA was observed. Immunohistochemistry and immunofluorescence analysis showed a higher proportion of O-GlcNAc-positive neurons in the NTG group, while O-GlcNAc colocalization with astrocytes/microglia was elevated in SOD1-G93A mice. Moreover, we reported the identification of 568 high-confidence O-GlcNAc sites from end-stage SOD1-G93A and NTG mice. Of the 568 sites, 226—many of which occurred on neuronal function and structure-related proteins—were found to be dynamically regulated.

**Conclusion:**

These data provide a valuable resource for dissecting the functional role of O-GlcNAcylation in ALS and shed light on promising therapeutic avenues for ALS. The chemoenzymatic labeling-based chemoproteomic approach is applicable for probing O-GlcNAc dynamics in various pathological processes.

**Supplementary Information:**

The online version contains supplementary material available at 10.1186/s10020-025-01134-4.

## Introduction

Amyotrophic lateral sclerosis (ALS), known as a rare but potentially fatal neurodegenerative disease, is characterized by progressive motor neuron loss in spinal cord, brainstem, and motor cortex, which leads to eventual paralysis and/or respiratory failure (Taylor et al. 2016; Xu et al. 2020). The average survival period of ALS patients after diagnosis is often three to five years. Riluzole and edaravone (Jaiswal 2019; Park et al. 2020), the FDA-approved drugs to treat ALS, are reported to extend patient survival by only a few months. To promote effective treatment for ALS, it is of vital importance to understand its pathogenesis. Emerging evidence suggests that dysregulation of O-linked-β-*N*-acetylglucosamine (O-GlcNAc, i.e. O-GlcNAcylation) is implicated in the pathological mechanism of ALS (Lüdemann et al. 2005; Shan et al. 2012).

O-GlcNAcylation is a ubiquitous post-translational modification in metazoans and occurs on serine (Ser, S) and/or threonine (Thr, T) residues of nucleocytoplasmic and mitochondrial proteins (Torres and Hart 1984). O-GlcNAc is regulated by only two opposite enzymes—the O-GlcNAc transferase (OGT) for linking a single GlcNAc onto Ser/Thr residues of protein substrates and the O-GlcNAcase (OGA) for removal of GlcNAc, making it reversible and dynamic (Yang and Qian 2017). O-GlcNAc has been found to modulate diverse biological processes (Chatham et al. 2021; Yang and Qian 2017), such as transcription, signal transduction, and protein homeostasis.

Although O-GlcNAc is ubiquitously expressed, it is highly abundant in brains. Loss of OGT in mouse forebrain excitatory neurons led to progressive neurodegeneration (Wang et al. 2016). Correlatively, aberrant O-GlcNAcylation is implicated in neurodegenerative diseases (Liu et al. 2009; Ryan et al. 2019), including ALS, Alzheimer’s disease (AD), and Parkinson’s disease (PD). Through a transgenic mouse model, Krieger and coworkers reported a lower O-GlcNAc level in the motor neurons of spinal cord from ALS mice (Shan et al. 2012). Moreover, a significant decrease of global O-GlcNAcylation was also observed in both brain tissues of AD subjects and experimental models (Liu et al. 2009; Pinho et al. 2019). These reduced O-GlcNAc events in AD showed strong correlation with mitochondrial anomalies (Pinho et al. 2019). In PD, elevation of O-GlcNAcylation facilitates alleviating neuroinflammation of the midbrain (Kim et al. 2024). Several regulators of neurodegenerative diseases are modulated by O-GlcNAc. For instance, O-GlcNAcylation of amyloid-β precursor protein (APP) impaired APP trafficking to the plasma membrane and thus attenuated pathological Aβ production in AD (Chun et al. 2017). It is of great interest to quantitatively profile the O-GlcNAc proteins and modification sites during the development of neurodegenerative diseases.

It should be noted that only a few O-GlcNAcylated proteomic analyses have been performed in the context of neurodegenerative disease progression. By combining chemoenzymatic labeling and mass spectrometry (MS)-based quantitative proteomics, the Liu and Gong group identified 131 altered O-GlcNAcylated peptides from human brain tissues with or without AD (Wang et al. 2017). However, to our best knowledge, large-scale proteomic profiling of O-GlcNAcylation in ALS pathogenesis is still unexplored.

Herein, we employ SOD-G93A mice and its non-transgenic (NTG) littermates, the most widely used animal model for studying ALS pathogenesis (Philips and Rothstein 2015), to characterize the dynamic changes of O-GlcNAcylation during ALS progression. We discover that the overall O-GlcNAc level is significantly reduced at the end stage of ALS compared to that in NTG, which might attribute to a great increase of OGA. Through a chemoenzymatic labeling-based chemoproteomic strategy, we report the identification of O-GlcNAcylation sites in the spinal cord from end-stage SOD1-G93A and NTG mice, giving a total of 568 high-confidence sites. The abundances of these O-GlcNAc sites are furthermore quantified. 215 are found to be down-regulated during ALS pathogenesis, many of which occur on neuronal function regulators. These results provide a valuable database for dissecting the regulatory role of O-GlcNAcylation in ALS disease.

## Materials and methods

### Animals

Male hemizygous transgenic mice (overexpressing human SOD1-G93A) purchased from Jackson Laboratories (B6SJL-Tg(SOD1-G93A)1Gur/J, Strain #: 002726) were mated with female B6SJL F1 mice to obtain transgenic human SOD1-G93A mice and their non-transgenic (NTG) littermates. As reported (Gurney et al. 1994), the genomic DNA of mouse tails was extracted and analyzed to determine their genotypes. All mice were housed in groups (4 ~ 5 mice per cage) under the same conditions (alternating light/dark for 12 h, room temperature (R.T.) at 24 °C, relative humidity for 50-60%, ad libitum access to food and water). Spinal cord from female SOD1-G93A mice is collected in the following stages: (1) pre-symptomatic stage (60 days after birth), in which mice exhibit no motor defects but have pathological changes, including mild gliosis and sparse degeneration of neurons; (2) onset stage (about 90 days after birth), in which mice exhibit visible leg trembling in tail suspension test. (3) end stage (about 130 days after birth), in which mice are unable to upright itself within 30s after they were placed on their back or sides. Female NTG littermates were sacrificed at the corresponding ages and used as control. All animal experiments were conducted in accordance with the Regulations on the Administration of Laboratory Animals issued by the Ministry of Science and Technology of the People’s Republic of China and approved by the Experimental Animal Ethics Committee of the Xuanwu Hospital of Capital Medical University. All mice were anaesthetized under 1.0–3.0% (vol/vol) isoflurane (RWD Life Science, Shenzhen, China).

### Specimen Preparation

Fresh specimen preparation: After anesthesia, mice at indicated disease stages were perfused transcardially with 0.9% saline. Lumbar spinal cord was blown out through the sacral foramen, frozen in liquid nitrogen, and stored at -80 °C for subsequent Western blot experiments.

Histological specimen preparation: Anesthetized mice at indicated disease stages were perfused transcardially with 0.9% saline, followed by 4% paraformaldehyde in phosphate buffer saline (PBS). Then the lumbar spinal cord was carefully dissected and fixed in 4% paraformaldehyde (PFA) for 24 h. After cryoprotection in 30% sucrose solution (4 °C, overnight), the resulting spinal cord was embedded in O.C.T. compound and stored at -80 °C. About 25-µm-thick lumbar spinal cord sections were cut on a Leica cryostat (CM1850) and subjected to *Immunohistochemistry (IHC)/Immunofluorescence (IF)*.

### Antibodies

Antibodies included mouse anti-O-GlcNAc (Abcam, London, UK, ab-2739, 1:1000 for WB, 1:100 for IHC and IF), rabbit anti-OGT (Abcam, London, UK, ab-177941, 1:1000 for WB, 1:100 for IHC and IF), rabbit anti-OGA (Boster, Wuhan, China, A32463, 1:1000 for WB, 1:100 for IHC and IF), mouse anti-GAPDH (Bioworld, Nanjing, China, MB001, 1:5000), mouse anti-NeuN (Millipore, MAB377, 1:100), mouse anti-GFAP (Millipore, MAB360, 1:500), mouse anti-Iba-1 (GeneTex, San Antonio, CA, USA, GTX632426, 1:100), rabbit anti-GFAP (Cell Signaling Technology, Danvers, MA, USA, mAb80788, 1:200), rabbit anti-Iba-1 (Wako Chemicals, Osaka, Japan, 019-19741, 1:250), biotin-coupled secondary antibody (Bioworld, Nanjing, China, MB001, 1:5000), Dylight 800 conjugated goat anti-mouse IgG (Abbkine, Wuhan, China, A23910, 1:5000), Dylight 800 conjugated goat anti-rabbit IgG (Abbkine, Wuhan, China, A23920, 1:5000), donkey anti-Rabbit IgG (H + L) Highly Cross-Adsorbed Secondary Antibody, Alexa Fluor™ 488 (Thermo, MA, USA, A21206, 1:1000), Alexa Fluor™ 568 (Thermo, MA, USA, A10037,1:1000), and Donkey anti-Mouse IgG (H + L) Highly Cross-Adsorbed Secondary Antibody.

### Western blot (WB)

Total proteins were extracted from frozen tissues by using a protein extraction kit (Beijing Solarbio Science and Technology Co., Ltd., China) according to the manufacturer instructions. The samples in loading buffer containing 10% SDS were denatured at 95 °C for 10 min, resolved on a 10% SDS-PAGE gel, and transferred onto pre-activated polyvinylidene fluoride (PVDF) membrane. Then the membrane was blocked by 5% skimmed milk and incubated with indicated primary antibodies at 4 °C, overnight. After washing with TBST for three times, the resulting membrane was incubated with Delight 800-conjugated anti-rabbit/mouse IgG for 2 h at R.T. and imaged by an Odyssey Infrared imaging system (LI-COR, Lincoln, NE, USA).

### Immunohistochemistry (IHC)

Lumbar spinal cord sections were treated with 3% hydrogen peroxide for 1 h, washed with PBS for three times, and permeabilized in 10% goat serum containing 0.3% TritonX-100 at R.T, followed by incubation with primary antibody overnight at 4 °C. The resulting sections were washed with PBS containing 0.2% Tween 20 (PBST) for three times and reacted with biotin-conjugated secondary antibody for 1 h. After washing with PBST, the sections were incubated with Vectastain ABC reagent (Vector Laboratories, Burlingame, CA, USA, PK-6100) and ImmPACT DAB Peroxidase Substrate Kit (Vector, SK-4105) for immunohistochemical staining. Then the reacted sections were placed on slides, and the resulting slides were soaked in anhydrous ethanol for 5 min and xylene for 10 min. After sealing with neutral gum, the slides were analyzed by an Olympus microscope (BX53) equipped with a DP73 CCD. For each group, five floating lumbar spinal cord sections from each mouse (*n* ≥ 3) were used for immunohistochemistry analysis.

### Immunofluorescence (IF)

Lumbar spinal cord sections were permeabilized with 1% Triton X-100 in PBS for 30 min and blocked with 5% goat serum in PBS containing 0.1% Triton X-100 for 30 min, followed by incubation with primary antibody overnight at 4 °C. After washing with PBS for three times, the resulting sections were incubated with the fluorescence-conjugated secondary antibody for 2 h at R.T., placed on slides, stained with DAPI (VECTOR, VECTASHIELD H-1200), and sealed with nail polish. Immunofluorescence imaging was performed on Olympus confocal microscope (FV1000). Images were acquired by the software Olympus Viewer 3. There are five mice in each group, and one floating lumbar spinal cord section per mouse was processed for immunofluorescence analysis.

### Chemoenzymatic labeling of protein O-GlcNAcylation

The protein lysates were extracted as mentioned above, followed by adjusting protein concentration into 2 mg/mL by BCA protein assay kit (Thermo). For every 1 mL protein solutions, 3 mL methanol, 750 µL chloroform, and 2 mL Milli-Q H_2_O were added. The resulting sample was then centrifugated at 12,000 *g* for 10 min at 4 °C. After removal of the aqueous phase, the protein pellets were washed with cold methanol twice and dissolved in 400 µL Hepes buffer (20 mM, pH 7.9) containing 1% SDS (wt/vol). Subsequently, 490 µL Milli-Q H_2_O, 800 µL labeling buffer [125 mM NaCl. 5% Nonidet P-40 (vol/vol), 50 mM Hepes, pH 7.9], 5.5 mM MnCl_2_, 0.025 mM UDP-GalNAz, and 75 µL Y289L GalT1 were added. In the negative control, the Y289L GalT1 was omitted. The mixture was at 4 °C for 24 h with gentle rotation. After methanol-chloroform-H_2_O precipitation, the reacted samples were resuspended with 1 mL of Hepes buffer (20 mM, pH 7.9) containing 0.5% SDS (wt/vol), and subjected to *click labeling.*

### Click labeling

For in-gel fluorescence scanning, 50 µL resuspensions were incubated with 300 µM CuSO_4_-BTTAA pre-mixed complex (CuSO_4_-BTTAA, 1:2 molar ratio), 100 µM alkyne-Cy5 (Click Chemistry Tools, TA116), and 2.5 mM fresh sodium ascorbate for 2 h at room temperature (R.T.). The resulting sample was then resolved on 10% SDS-PAGE. The gel was imaged by Typhoon FLA 9500 (GE) and then stained by Coomassie Brilliant Blue (CBB) to demonstrate equal loading. For chemoproteomic profiling of O-GlcNAcylation sites, spinal cord lysates from four SOD1-G93A or NTG mice were processed for one biological replicate. As described above, 1.5 mL resuspensions of spinal cord lysates from SOD1-G93A mice (or its non-transgenic littermates) were incubated with 300 µM CuSO_4_-BTTAA pre-mixed complex (CuSO_4_-BTTAA, 1:2 molar ratio), 100 µM alkyne-H-PC-biotin (or alkyne-L-PC-biotin), and 2.5 mM fresh sodium ascorbate for 2 h at R.T. Then the isotope-labeled samples were combined, precipitated, and resuspended with 600 µL 8 M urea in H_2_O, followed by *Enrichment of O-GlcNAcylated peptides and UV cleavage*.

### Enrichment of O-GlcNAcylated peptides and UV cleavage

The protein solution was diluted into 4 M urea with 100 mM ammonium bicarbonate (ABC) in H_2_O, followed by treatment with 10 mM dithiothreitol (45 min, 37 °C) and 20 mM iodoacetamide (30 min, in dark, R.T.). Then the reacted sample was diluted to 0.8 M urea with 50 mM ABC and incubated with trypsin (enzyme-to-substrate ratio, 1:50) for 16 h at 37 °C, followed by addition of 150 µL streptavidin beads (Thermo, 20353) with gentle rotation for 4 h at R.T. The resulting beads were washed with PBS (pH 7.4) for six times and Milli-Q water for six times. Then the beads were resuspended in 300 µL 0.1% formic acid (in H_2_O) and irradiated with UV light (365 nm) for a total of 30 min by a UV cross-linker (UVP, CL-100). After centrifugation to remove streptavidin beads, the released O-GlcNAc peptides were evaporated in a speed vacuum centrifuge and subjected to *LC-MS/MS*.

### LC-MS/MS

MS raw data were generated from an Orbitrap Fusion Lumos mass spectrometer (Thermo) fitted with a Dionex Extreme 3000 RPLC nano system (Thermo). The peptides were dissolved in 0.1% formic acid (in H_2_O) and separated by a EasySpray reversed-phase LC column (75 μm × 50 cm) packed with PepMap C18 particles (100 Å, 2 μm). The mobile phases (A: 0.1% formic acid; B: 0.1% formic acid in 80% acetonitrile) were used and the LC gradient was: 1% B for the first 8 min, 1–7% B in 1 min, 7–35% B from 9 min to 311 min, 35–44% B from 311 min to 353 min, and 44–99% B from 353 min to 356 min. Full MS scans of peptide precursors (350–2000 Th) were acquired with a resolution of 120,000, a maximum injection time of 50 ms, and RF lens at 60%. The multicharged precursors (z = 2–8) were selected for MS/MS scans with a cycle time of three seconds, and monoisotopic precursor selection was enabled. High-energy collisional dissociation product-dependent electron-transfer/higher-energy collisional dissociation (HCD pd EThcD) was employed for MS/MS fragmentation. If one of the glycan oxonium ions (m/z 168.0655, 186.0761, 204.0865, 274.0921, 292.1027, 300.1302, 329.1455, 366.1395, 388.1463, 399.1992, and 405.213) was produced in a HCD scan, EThcD acquisition was further performed on the captured fragment.

### Data processing

MS data were processed with the software MaxQuant (Cox and Mann 2008) (version 1.6.1.0) integrated with Andromeda search engine. SwissPort *Mus musculus* proteome database downloaded from Uniprot on 4th November, 2016. As reported (Hao et al. 2023; Liu et al. 2022), modifications on Ser and Thr (601.2708 and 607.2846 Da for “light” and “heavy” O-GlcNAc-GalNAz tagged adducts by isoPTOP labeling) were searched as variable modifications separately. Moreover, O-HexNAc-containing peptides with a false discovery rate at 1% were filtered by an Andromeda score > 40 and a delta score > 8. O-HexNAc modification sites with a localization probability > 0.75 on the glycopeptides were defined as identified sites. By using the term “Subcellular Location” in Uniprot as a reference standard, extracellular O-HexNAc sites were excluded from the O-GlcNAc site list. O-GlcNAc sites identified at least twice were defined as high-confidence sites. Site-quantification was processed with the software CIMAGE (Gao et al. 2021; Liu et al. 2022). Briefly, extracted MS1 ion chromatograms (± 10 ppm) of the coeluting isotope-modified peptides were generated using a ± 10-min retention time window and the corresponding “light”/“heavy” ratio was calculated. The median value of all modified peptides was used as the final quantification ratio of the O-GlcNAc site. High-confidence sites quantified twice or more were analyzed for enrichment significance.

### Statistical analysis

Student’s t-tests were used to calculate significant differences between two groups. Experiments include 3–8 mice per group. Data results are expressed as mean ± standard deviation (mean ± SD), and *P value* < 0.05 indicates statistically significant differences. For comparing the enrichment significance of O-GlcNAc sites between SOD1-G93A and NTG mice, *P values* were calculated by a Bayes moderated t-test and adjusted by the Benjamini-Hochberg method.

## Results

### Reduced level and altered cellular location of O-GlcNAcylation in lumbar spinal cord of SOD1-G93A mice

**Fig. 1:**
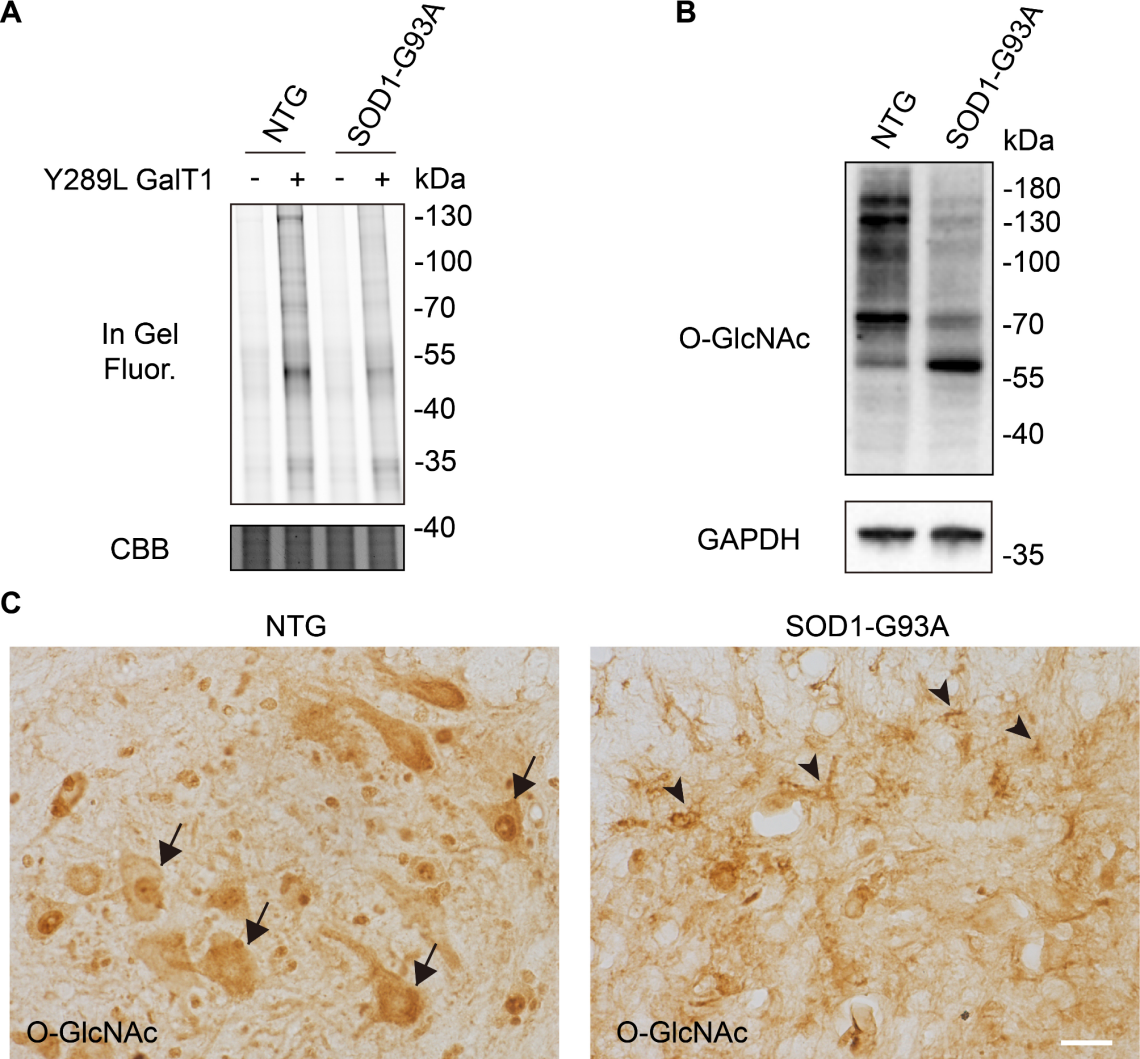
Reduced O-GlcNAc levels in lumbar spinal cord of SOD1-G93A mice. **(A)** In-gel fluorescence scanning showing lumbar spinal cord lysates of SOD1-G93A at end stage and nontransgenic (NTG) mice. The cell lysates were chemoenzymatically labeled and reacted with alkyne-Cy5 via CuAAC. Coomassie Brilliant Blue (CBB)-staining demonstrates comparable loading. Representative results are from three independent experiments. **(B)** Immunoblotting showing the overall O-GlcNAc level of lumbar spinal cord from SOD1-G93A at end stage and NTG mice. RL2 is an O-GlcNAc-recognizing antibody. Anti-GAPDH blot demonstrates comparable loading. Representative results are from three independent experiments. **(C)** Representative immunohistochemistry staining images of O-GlcNAcylation in lumbar spinal cord anterior horn of NTG and end-stage SOD1-G93A mice. Arrows indicate O-GlcNAc-positive motor neurons. Arrowheads indicate O-GlcNAc-positive glial cells. Representative images are from three independent experiments. Scale bars, 50 μm

We sought to investigate the dynamic changes of O-GlcNAcylation in three distinct disease stages during ALS progression: pre-symptomatic stage (about 60 days after birth), onset stage (about 90 days after birth), and end stage (about 130 days after birth). The lumbar spinal cord of NTG and SOD1-G93A mice at the indicated stages was collected and lysed, followed by chemoenzymatically labeling with UDP-*N*-azidoacetylgalactosamine (UDP-GalNAz) and Y289L GalT1 (a mutant galactosyltransferase). The Y289L GalT1 specifically tags terminal GlcNAc with a GalNAz moiety by using UDP-GalNAz as the sugar donor (Clark et al. 2008). Then the reacted lysates were conjugated with alkyne-Cy5 via Cu(I)-catalyzed azide−alkyne cycloaddition (CuAAC, or click chemistry) (Uttamapinant et al. 2012) and the labeling of O-GlcNAcylated proteins were analyzed by in-gel fluorescence scanning. Of note, the overall O-GlcNAc level was significantly decreased at disease end stage compared to that in NTG (Fig. 1A and Fig. S1-S3). Similar results were observed in western blot analysis by using an O-GlcNAc-recognizing antibody anti-RL2 (Fig. 1B and Fig. S3).

**Fig. 2:**
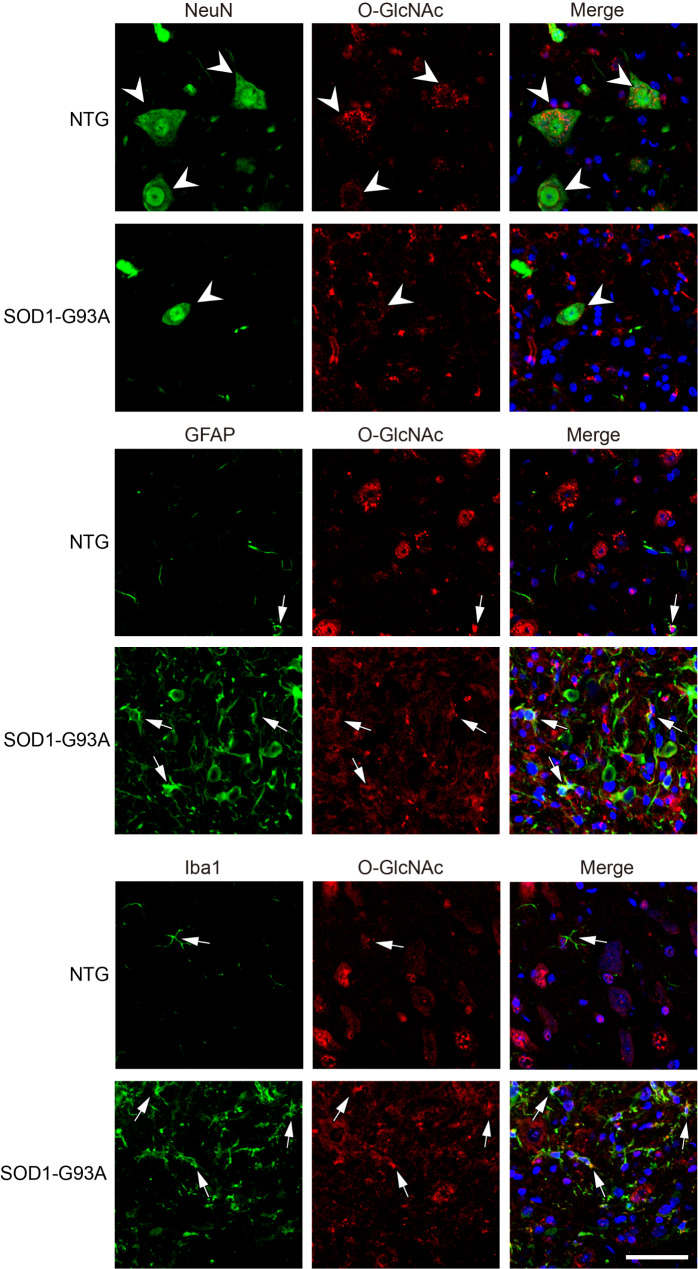
Characterizing cellular O-GlcNAc distribution in lumbar spinal cord of SOD1-G93A and NTG mice. Confocal fluorescence images of O-GlcNAc-NeuN/GFAP/Iba1 colocalization in lumbar spinal cord cells from SOD1-G93A at the end stage and NTG mice. NeuN, a neuronal marker. GFAP, an astrocyte-specific marker. Iba1, a microglia marker. Arrowheads indicate colocalization of NeuN and O-GlcNAc. Arrows indicate colocalization of GFAP/Iba1 with O-GlcNAc. Representative images are from five independent experiments. Scale bars, 50 μm

To investigate the subcellular location of O-GlcNAcylation, lumbar spinal cord from NTG and SOD1-G93A mice were immunohistochemically stained by anti-RL2 antibody. Strong O-GlcNAc immunostaining was observed mostly in neuronal cell bodies and neurites in the NTG group (Fig. 1C), which was further proved in double-labelling immunofluorescence microscopy analysis (Fig. 2 and Fig. S4); while the SOD1-G93A group showed a dramatic increase of O-GlcNAc immunoreactivity in glial cells accompanied by an obvious reduction of O-GlcNAc positive neurons (Fig. 1C). As assayed by double-labelling immunofluorescence, the increased glial O-GlcNAcylation was mainly colocalized with proliferating microglia, as well as astrocytes, in lumbar spinal cord of SOD1-G93A mice (Fig. 2 and Fig. S4). The results indicate that O-GlcNAcylation is dynamically regulated in the development of ALS.

### Change of OGT and OGA expression in lumbar spinal cord of SOD1-G93A mice

**Fig. 3:**
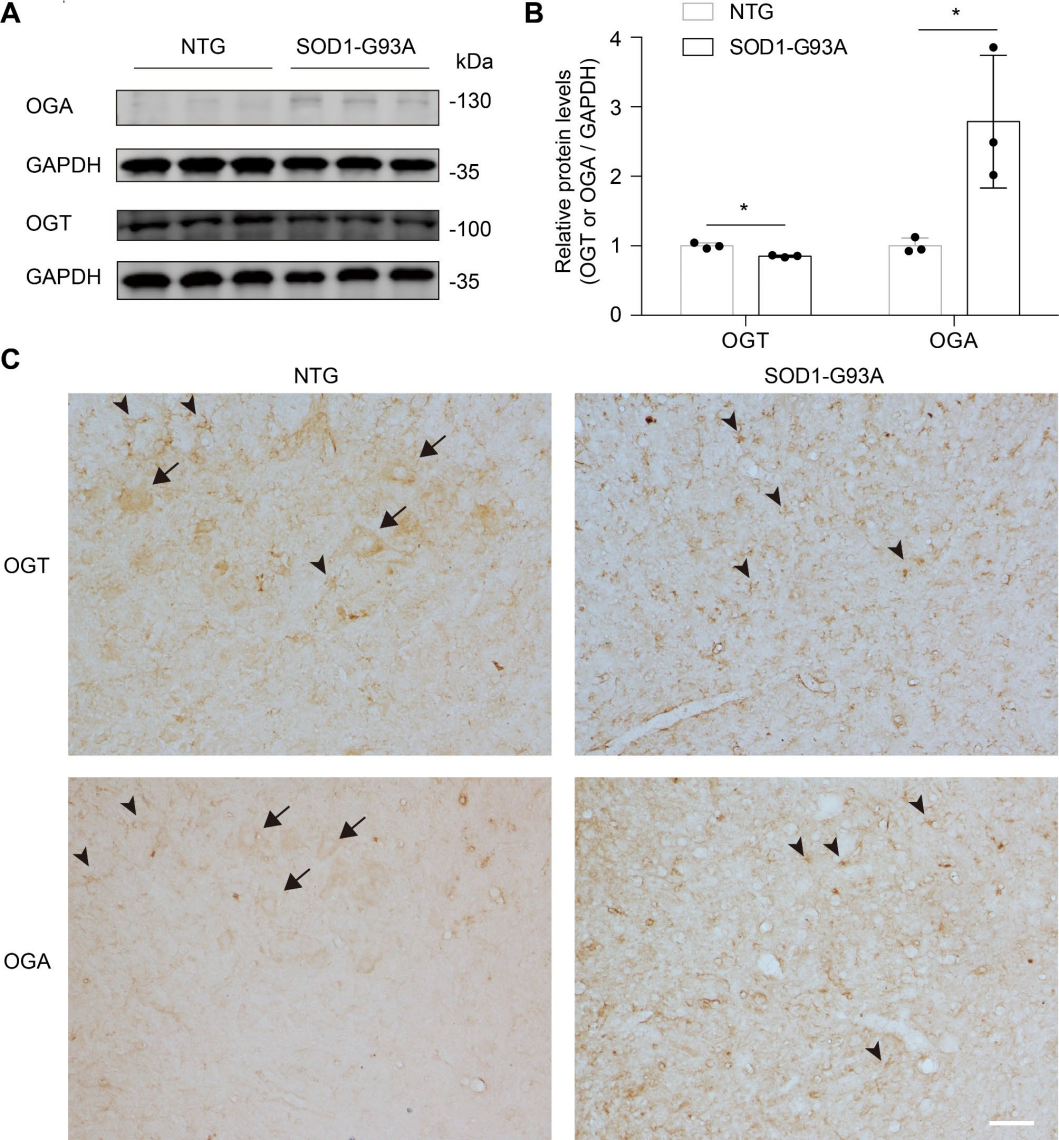
Altered expression of OGT and OGA in lumbar spinal cord of SOD1-G93A mice. (**A**) Immunoblots comparing the expression of OGT and OGA in lumbar spinal cord from SOD1-G93A at end stage and NTG mice. Anti-GAPDH blot demonstrates comparable loading. Results are from six independent experiments. **(B)** Bar graph showing statistical analysis of the relative protein level in **(A)**. *P < 0.05 (Student’s t-test). **(C)** Representative immunohistochemistry staining images of OGT/OGA in lumbar spinal cord anterior horn of NTG and end-stage SOD1-G93A mice. Arrows indicate OGT/OGA-positive motor neurons. Arrowheads indicate OGT/OGA-positive glial cells. Representative images are from three independent experiments. Scale bars, 50 μm

**Fig. 4:**
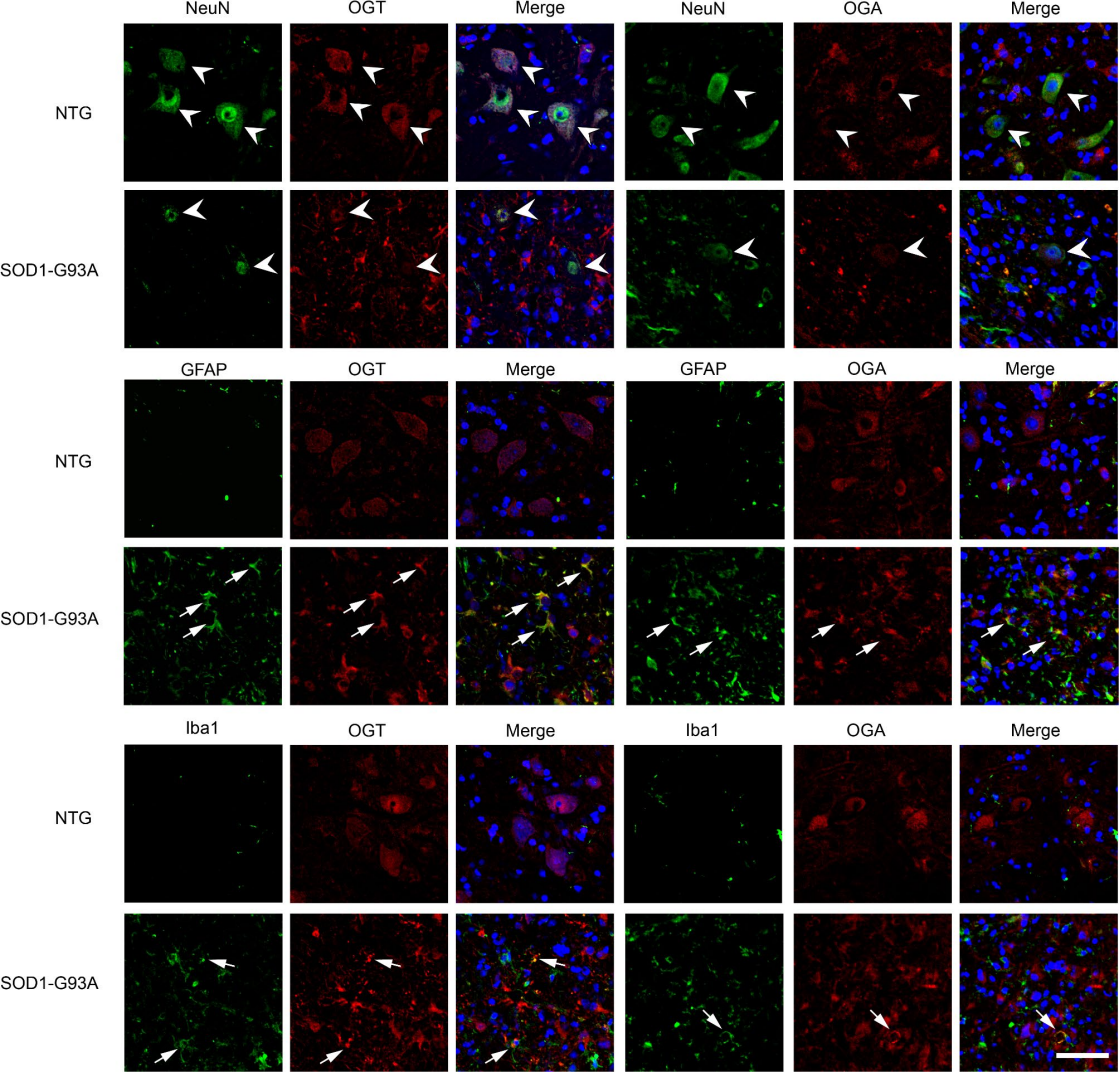
Characterizing cellular OGT/OGA distribution in lumbar spinal cord of SOD1-G93A and NTG mice. Confocal fluorescence images of OGT/OGA-NeuN/GFAP/Iba1 colocalization in lumbar spinal cord cells from SOD1-G93A at end stage and NTG mice. Arrowheads indicate colocalization of NeuN with OGT/OGA. Arrows indicate colocalization of GFAP/Iba1 with OGT/OGA. Representative images are from five independent experiments. Scale bars, 50 μm

We next asked the protein level of OGT/OGA between NTG and SOD1-G93A mice. Immunoblotting using an anti-OGA/OGT antibody showed a significant increase of OGA and a minor decrease of OGT in lumbar spinal cord of SOD1-G93A mice at the end stage (Fig. 3A, B and Fig. S5), which contributed to a reduced O-GlcNAc level during ALS progression, respectively. To examine the cellular localization of OGT/OGA, immunohistochemistry and double-labelling immunofluorescence staining were then performed. In normal lumbar spinal cord of the NTG group, OGT/OGA immunoreactivity was mainly distributed in the cytoplasm of neurons and glia; Of note, in spinal cord of SOD1-G93A mice, OGT/OGA-positive neurons were dramatically declined but the OGT/OGA-GFAP or -Iba1 co-localized astrocytes and microglia were increased. (Figures 3C and 4 and Fig. S6). These results suggest that the expression of OGT/OGA is modulated in an ALS pathogenesis-dependent manner.

### Quantitative and site-specific profiling of protein O-GlcNAcylation in lumbar spinal cord of NTG and SOD1-G93A mice

**Fig. 5:**
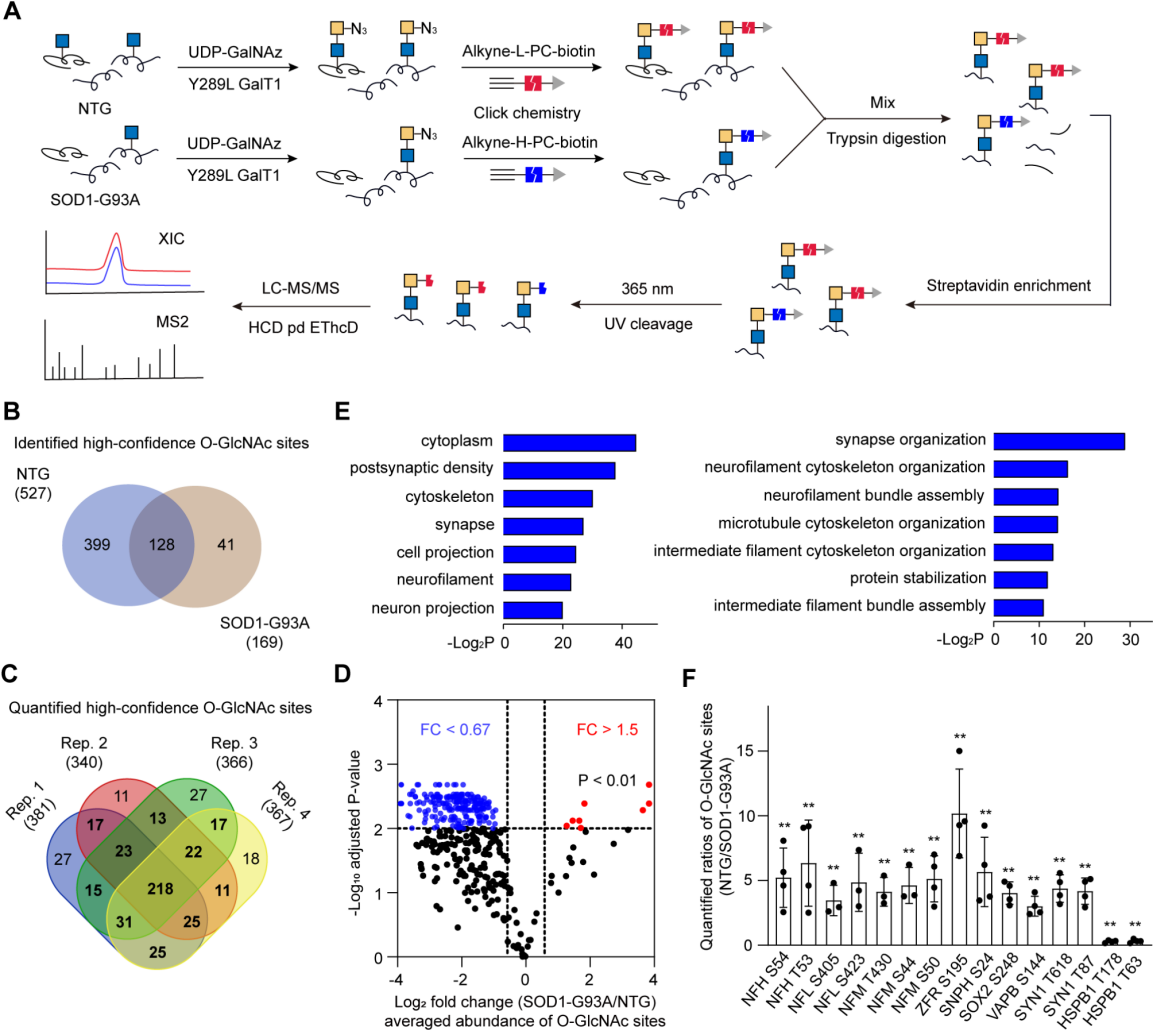
Quantitative and site-specific profiling of O-GlcNAcylation in NTG and SOD1-G93A mice. **(A)** Workflow of large-scale identification and quantification of O-GlcNAc sites between NTG and end-stage SOD1-G93A mice by isoPTOP. Results are from four independent experiments. **(B)** Overlap of high-confidence O-GlcNAc sites identified in NTG and SOD1-G93A mice. **(C)** Overlap of quantified high-confidence O-GlcNAc sites between NTG and SOD1-G93A mice. **(D)** Volcano plot showing the relative abundance of O-GlcNAcylation sites in NTG and SOD1-G93A mice. The blue dots (fold change < 0.67 and adjust *P* value < 0.01) represent enriched sites in NTG, and the red dots (fold change > 1.5 and adjust P value < 0.01) are enriched sites in SOD1-G93A. **(E)** Cellular Component and Biological Process GO terms of proteins harboring O-GlcNAc sites enriched in NTG. **(F)** Bar graph showing the relative abundance of O-GlcNAcylation sites on several neuronal function and structure related regulators. In (**D**) and (**F**), *P* values were adjusted by the Benjamini-Hochberg method. **P < 0.01

We then employed the isotopic photocleavable tagging strategy for O-GlcNAc profiling (isoPTOP) (Hao et al. 2023; Liu et al. 2022) to quantitatively profile O-GlcNAcylation in site-specific manner between NTG and SOD1-G93A mice at disease end stage. After chemoenzymatic labeling, the lumbar spinal cord lysates of NTG and SOD1-G93A mice were click-labeled with “light” alkyne-biotin containing a photocleavable linker (alkyne-L-PC-biotin) and “heavy” alkyne-PC-biotin (alkyne-H-PC-biotin), respectively (Fig. 5A and Fig. S7). The biotinylated cell lysates were combined, trypsin-digested, and captured by streptavidin beads. After UV cleavage, the released glycopeptides were analyzed by LC-MS/MS.

In agreement with the previous studies (Hao et al. 2023), a streamlined procedure was applied to define identified and quantified O-GlcNAcylation sites (Fig. S8A). In four biological replicates, a total of 894 and 396 O-GlcNAcylation sites were identified in NTG and SOD1-G93A mice (Fig. S8B, S8C, S9, S10, and Table S1), giving 527 and 169 high-confidence O-GlcNAcylation sites, respectively (Fig. 5B). Of these high-confidence sites, 417 were quantified at least twice (Fig. 5C and Table S2). Of the 417 quantified sites, 215 exhibited a significant down-regulation in SOD1-G93A compared to those in NTG mice, while 11 sites were enriched in SOD1-G93A mice (Fig. 5D). Gene ontology (GO) analysis indicated that the 215 down-regulated O-GlcNAc events were mostly involved in neuronal functions (Fig. 5E), such as synapse organization, neurofilament cytoskeleton organization, and neurofilament bundle assembly. It should be noted that the anti-RL2 blot in SOD1-G93A mice shows a stronger immunoreactive band (above 55 KDa) (Fig. 1B). Consistently, the increase of T110 O-GlcNAcylation on PDLIM5 (~ 63 KDa) was identified in SOD1-G93A mice (Fig. 5D and Table S2). These results, to some extent, further supports our finding in ALS pathogenesis.

O-GlcNAcylation on a series of neuronal function and structure related proteins was altered in SOD1-G93A mice (Fig. 5F). For instance, Neurofilament (NF) is regarded as an essential axonal cytoskeleton component, which consists of NF-heavy (NFH), NF-medium (NFM), and NF-light (NFL) (Geisler et al. 1983; Yuan et al. 2017). Our results showed that NF was heavily O-GlcNAcylated in NTG mice, while its O-GlcNAcylation level was reduced in the SOD-G93A group. Given that O-GlcNAc alleviates NF pathological aggregation (Peng et al. 2019), we speculate a profound defect of NF structure and function at the end stage of ALS. HSPB1 (also referred to as HSP27) mediates neuroprotection in neurodegenerative disorders by inhibiting protein misfolding and accumulation (Cox et al. 2018; Yang et al. 2024). O-GlcNAcylation of HSPB1 was reported to facilitate refolding of protein substrates via protein-protein interactions with the BAG3/HSP70 co-chaperone (Javed et al. 2024). We found that HSPB1 was O-GlcNAcylated in ALS pathogenesis. Moreover, HSPB1 O-GlcNAcylation at Thr 178/Thr 63 exhibited approximately 3.5/3.2-fold higher in SOD-G93A than NTG mice, suggesting a potential neuroprotective reaction in ALS disease. O-GlcNAcylation of Synapsin 1 (SYN1) at Thr 87 suppresses SYN1 localization to synapses and then impairs the formation of the reserve pool of synaptic vesicles, thus modulating presynaptic plasticity in mature neurons (Skorobogatko et al. 2014). Interestingly, the abundance of O-GlcNAcylation on SYN1 was significantly decreased in SOD-G93A mice, which indicates a possible link between defective synaptic functions and ALS progression.

## Discussion

O-GlcNAcylation protects against pathological protein aggregation and misfolding in neurodegenerative diseases (Peng et al. 2019; Ryan et al. 2019). We discovered the overall O-GlcNAcylation was significantly decreased at the end stage of ALS transgenic mice. In consistence with this result, an elevated level of OGA was observed. The findings suggest that OGA inhibition might be a potential therapeutic strategy for ALS treatment. Of note, Thiamet-G and its derivative MK-8719, two OGA inhibitors that can cross the blood-brain barrier in vivo (Rostgaard et al. 2023; Wang et al. 2020), have been proven to be effective for the treatment of tauopathies. It will be of great interest to evaluate the clinical use of Thiamet-G and MK-8719 in ALS.

O-GlcNAcylation plays an important role in the central nervous system (CNS), both during development and in the process of CNS diseases (Zhang et al. 2024). OGT deletion in adult mice forebrain caused progressive neurodegeneration (Wang et al. 2016). Our immunohistochemistry and immunofluorescence analysis suggests dramatic loss of O-GlcNAc-positive neurons in ALS transgenic mice at the end disease stage, which correlates with the profound downregulation of O-GlcNAcylation sites on neuronal structure and function proteins. Meanwhile, the reduction of O-GlcNAcylation in motor neurons of G93A lumbar spinal cords appears to be partially balanced by increased O-GlcNAc in astrocytes and microglia. From immunohistochemistry images, we can observe an overall O-GlcNAc/OGT/OGA expression profile and shift of positive-expressing cell types. Double-labelling immunofluorescent assays provide cellular localization information in particular locations (anterior horn of spinal cord) at higher magnifications. However, these two histological methods can only to some extent indicate expression changes, but they are hard to quantify the overall expression changes in the spinal cord as Western blot did. Nevertheless, these three methods are complementary to and in most cases in consistent with each other. From double immunofluorescence labelling, we observed an increase of OGT/OGA expression in reactive astrocytes and an elevated O-GlcNAc level in activated microglia in SOD1-G93A mice. It should be noted that astrocytic O-GlcNAcylation was reported to inhibit its activation and inflammation (Dong et al. 2023), nonetheless, the exact role of O-GlcNAc in microglia remains to be explored.

Quantitative and site-specific profiling of O-GlcNAcylation is a prerequisite for understanding O-GlcNAc regulatory roles during ALS progression. By using isoPTOP, we identified 527 and 169 high-confidence O-GlcNAcylation sites in end stage SOD1-G93A and NTG mice, respectively. Of the identified sites, 215 were down-regulated in SOD1-G93A mice, many of which occurred on neuronal structure and function regulators. For example, O-GlcNAcylation of SYN1 at Thr 87 was decreased during ALS progression. SYN1 O-GlcNAcylation has been reported to modulate presynaptic plasticity (Skorobogatko et al. 2014). It is interesting to speculate correlations between synaptic dysregulation and ALS pathogenesis. Of note, considering that O-GlcNAc has been reported to stabilize a series of protein substrates (Hao et al. 2019; King et al. 2022; Makwana et al. 2019), it is worthy to address whether this is a generic effect of O-GlcNAc on its modified proteins involved in ALS pathogenesis.

Phosphorylation as one of the best characterized post-translational modifications (PTMs) can decorate numerous proteins and modulate their biological functions (Battaglioni et al. 2022; Singh et al. 2017). Akin to phosphorylation, O-GlcNAcylation occurs on Ser and Thr residues of protein substrates. O-GlcNAc has been found to disrupt phosphorylation by competition for the same site(s) and/or by modifications influencing residue(s) in proximity on the protein sequence, which is involved in co-regulation of protein functions (Hart et al. 2011; Leney et al. 2017). In this context, further studies are needed to fully elucidate the crosstalk between O-GlcNAcylation and phosphorylation.

**Fig. 6:**
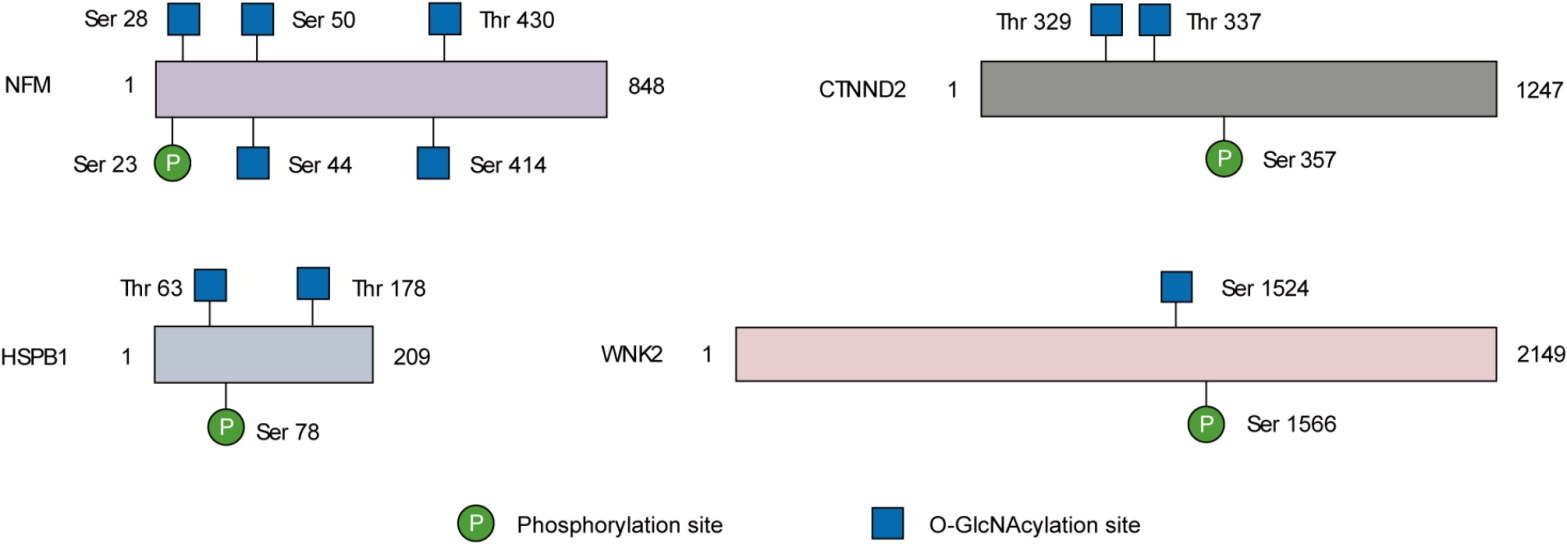
Potential O-GlcNAcylation-phosphorylation crosstalk on specific proteins involved in regulation of neuronal functions. Schematic diagram showing O-GlcNAcylation sites and adjacent phosphorylation sites on neurofilament medium (NFM), Heat shock protein HSPB1, synaptic protein Catenin delta-2 (CTNND2), and protein kinase WNK2

Several neuronal function-related proteins carrying potential crosstalk between O-GlcNAcylation and phosphorylation were identified in our work. For example, NFM was O-GlcNAcylated at Ser 28, Ser 44, and Ser 50 (Fig. 6), which are adjacent to the phosphorylation site Ser 23 (Sihag et al. 1997). A decrease of O-glycosylation along with enhanced phosphorylation on NFM has been reported in a transgenic rat model of ALS (Lüdemann et al. 2005). It is interesting to address crosstalk between O-GlcNAc and phosphorylation in the context of ALS pathogenesis. Phosphorylation of the synaptic protein Catenin delta-2 (CTNND2) governs GluR2-mediated synaptic activity (Poore et al. 2010). In proximity to the phosphorylated residue, Thr 329 and Thr 337, two O-GlcNAc sites of CTNND2, were identified in this study. HSPB1, a heat shock protein that promotes neuronal survival (Yang et al. 2024), is modified by both O-GlcNAc and phosphorylation (Netsirisawan et al. 2018; Song et al. 2019). In hepatoblastoma, it was reciprocally regulated by the two PTMs (Song et al. 2019). Notably, the abundance of O-GlcNAcylation on HSPB1 was significantly elevated in SOD1-G93A mice. This O-GlcNAc-phosphorylation crosstalk event may regulate the development of ALS. WNK2, a neuron-enriched kinase, was identified to be O-GlcNAcylated at Ser 1524. Moreover, it exhibited lower O-GlcNAcylation levels at the end stage of ALS compared to those of NTG. Phosphorylation of WNK2 at Ser 1566 can modulate γ-aminobutyric acid (GABA)-ergic neurotransmission in the mouse brain (Rinehart et al. 2011). The crosstalk between O-GlcNAcylation and phosphorylation on these residues remains to be explored, which probably provides a new molecular mechanism for regulation of WNK2 functions.

## Conclusion

In summary, we analyzed the dynamic changes of O-GlcNAcylation during ALS progression. The global O-GlcNAcylation is dramatically decreased at the end stage of ALS transgenic mice, and the reduced O-GlcNAc level might be mainly attributed to an obvious increase of OGA. Correlatively, cellular localization analysis manifested dramatic neuronal downregulation and glial upregulation of colocalized O-GlcNAcylation and OGT/OGA expression. We further applied isoPTOP (Hao et al. 2023; Liu et al. 2022) to compare the difference of O-GlcNAcylation sites between SOD1-G93A and NTG mice in quantitative manner, giving a total of 215 down-regulated sites in SOD-G93A mice. As assayed by GO analysis, these dysregulated O-GlcNAc events are mostly involved in neuronal structure and functions. Our results provide an invaluable resource for deciphering biological functions of O-GlcNAc in ALS pathogenesis. We envision that the chemoproteomic method should be generally applicable for probing O-GlcNAc dynamics in various pathological processes.

## Electronic supplementary material

Below is the link to the electronic supplementary material.

Supplementary Material 1

Supplementary Material 2

Supplementary Material 3

## Data Availability

The mass spectrometry proteomics raw data have been deposited to the ProteomeXchange Consortium via the PRIDE partner repository with the dataset identifier PXD059664. The other data used and/or analyzed during the current study are available from the corresponding author on reasonable request.

## Abbreviations

ALS: Amyotrophic lateral sclerosis
O-GlcNAc: O-linked β-*N*-acetylglucosamine
NTG: Non-transgenic
Ser: S Serine
Thr: T Threonine
OGT: The O-GlcNAc transferase
OGA: The O-GlcNAcase
AD: Alzheimer’s disease
PD: Parkinson’s disease
APP: Amyloid-β precursor protein
MS: Mass spectrometry
PFA: Paraformaldehyde
IHC: Immunohistochemistry
IF: Immunofluorescence
UDP-GalNAz: UDP-*N*-azidoacetylgalactosamine
CuAAC: Cu(I)-catalyzed azide−alkyne cycloaddition
isoPTOP: The isotopic photocleavable tagging strategy for O-GlcNAc profiling
NFH: Neurofilament-heavy
NFM: Neurofilament-medium
NFL: Neurofilament-light
PTMs: Post-translational modifications
CNS: Central nervous system
CBB: Coomassie Brilliant Blue

## References

[CR1] Battaglioni S, Benjamin D, Wälchli M, Maier T, Hall MN. mTOR substrate phosphorylation in growth control. Cell. 2022;185:1814–36. 35580586 10.1016/j.cell.2022.04.013

[CR2] Chatham JC, Zhang J, Wende AR. Role of O-linked *N*-acetylglucosamine protein modification in cellular (patho)physiology. Physiol Rev. 2021;101:427–93. 32730113 10.1152/physrev.00043.2019 PMC8428922

[CR3] Chun YS, Kwon OH, Chung S. O-GlcNAcylation of amyloid-β precursor protein at threonine 576 residue regulates trafficking and processing. Biochem Biophys Res Co. 2017;490:486–91. 10.1016/j.bbrc.2017.06.067 28624365

[CR4] Clark PM, Dweck JF, Mason DE, Hart CR, Buck SB, Peters EC, Agnew BJ, Hsieh-Wilson LC. Direct in-gel fluorescence detection and cellular imaging of O-GlcNAc-modified proteins. J Am Chem Soc. 2008;130:11576–7. 18683930 10.1021/ja8030467 PMC2649877

[CR6] Cox J, Mann M. MaxQuant enables high peptide identification rates, individualized p.p.b.-range mass accuracies and proteome-wide protein quantification. Nat Biotechnol. 2008;26:1367–72. 19029910 10.1038/nbt.1511

[CR5] Cox D, Whiten DR, Brown JWP, Horrocks MH, San Gil R, Dobson CM, Klenerman D, van Oijen AM, Ecroyd H. The small heat shock protein Hsp27 binds α-synuclein fibrils, preventing elongation and cytotoxicity. J Biol Chem. 2018;293:4486–97. 29382725 10.1074/jbc.M117.813865 PMC5868268

[CR7] Dong X, Shu L, Zhang J, Yang X, Cheng X, Zhao X, Qu W, Zhu Q, Shou Y, Peng G, et al. *Ogt*-mediated O-GlcNAcylation inhibits astrocytes activation through modulating NF-κB signaling pathway. J Neuroinflammation. 2023;20:146. 37349834 10.1186/s12974-023-02824-8 PMC10286367

[CR8] Gao J, Liu Y, Yang F, Chen X, Cravatt BF, Wang C. CIMAGE2.0: an expanded tool for quantitative analysis of activity-based protein profiling (ABPP) data. J Proteome Res. 2021;20:4893–900. 34495668 10.1021/acs.jproteome.1c00455

[CR9] Geisler N, Kaufmann E, Fischer S, Plessmann U, Weber K. Neurofilament architecture combines structural principles of intermediate filaments with carboxy-terminal extensions increasing in size between triplet proteins. EMBO J. 1983;2:1295–302. 10872323 10.1002/j.1460-2075.1983.tb01584.x PMC555275

[CR10] Gurney ME, Pu H, Chiu AY, Dal Canto MC, Polchow CY, Alexander DD, Caliendo J, Hentati A, Kwon YW, Deng HX. Motor neuron degeneration in mice that express a human Cu,Zn superoxide dismutase mutation. Science. 1994;264:1772–5. 8209258 10.1126/science.8209258

[CR11] Hao Y, Fan X, Shi Y, Zhang C, Sun DE, Qin K, Qin W, Zhou W, Chen X. Next-generation unnatural monosaccharides reveal that ESRRB O-GlcNAcylation regulates pluripotency of mouse embryonic stem cells. Nat Commun. 2019;10:4065. 31492838 10.1038/s41467-019-11942-y PMC6731260

[CR12] Hao Y, Li X, Qin K, Shi Y, He Y, Zhang C, Cheng B, Zhang X, Hu G, Liang S, et al. Chemoproteomic and transcriptomic analysis reveals that O-GlcNAc regulates mouse embryonic stem cell fate through the pluripotency network. Angew Chem Int Ed. 2023;62:e202300500. 10.1002/anie.202300500 36852467

[CR13] Hart GW, Slawson C, Ramirez-Correa G, Lagerlof O. Cross talk between O-GlcNAcylation and phosphorylation: roles in signaling, transcription, and chronic disease. Annu Rev Biochem. 2011;80:825–58. 21391816 10.1146/annurev-biochem-060608-102511 PMC3294376

[CR14] Jaiswal MK. Riluzole and Edaravone: A Tale of two amyotrophic lateral sclerosis drugs. Med Res Rev. 2019;39:733–48. 30101496 10.1002/med.21528

[CR15] Javed A, Johnson OT, Balana AT, Volk RF, Langen A, Ahn BS, Zaro BW, Gestwicki JE, Pratt MR. O-GlcNAc modification of HSP27 alters its protein interactions and promotes refolding of proteins through the BAG3/HSP70 co-chaperone. Protein Sci. 2024;33:e5173. 39291732 10.1002/pro.5173 PMC11409196

[CR16] Kim DY, Kim SM, Cho EJ, Kwak HB, Han IO. Protective effect of increased O-GlcNAc cycling against 6-OHDA induced Parkinson’s disease pathology. Cell Death Dis. 2024;15:287. 38654003 10.1038/s41419-024-06670-1 PMC11039476

[CR17] King DT, Serrano-Negrón JE, Zhu Y, Moore CL, Shoulders MD, Foster LJ, Vocadlo DJ. Thermal proteome profiling reveals the O-GlcNAc-dependent meltome. J Am Chem Soc. 2022;144:3833–42. 35230102 10.1021/jacs.1c10621 PMC8969899

[CR18] Leney AC, Atmioui, El D, Wu W, Ovaa H, Heck AJR. Elucidating crosstalk mechanisms between phosphorylation and O-GlcNAcylation. Proc. Natl. Acad. Sci. U. S. A. 2017;114:E7255–E7261. 10.1073/pnas.1620529114 PMC5584407 28808029

[CR19] Liu F, Shi J, Tanimukai H, Gu J, Gu J, Grundke-Iqbal I, Iqbal K, Gong CX. Reduced O-GlcNAcylation links lower brain glucose metabolism and Tau pathology in Alzheimer’s disease. Brain. 2009;132:1820–32. 19451179 10.1093/brain/awp099 PMC2702834

[CR20] Liu J, Hao Y, Wang C, Jin Y, Yang Y, Gu J, Chen X. An optimized isotopic photocleavable tagging strategy for site-specific and quantitative profiling of protein O-GlcNAcylation in colorectal cancer metastasis. ACS Chem Biol. 2022;17:513–20. 35254053 10.1021/acschembio.1c00981

[CR21] Lüdemann N, Clement A, Hans VH, Leschik J, Behl C, Brandt R. O-glycosylation of the tail domain of neurofilament protein M in human neurons and in spinal cord tissue of a rat model of amyotrophic lateral sclerosis (ALS). J Biol Chem. 2005;280:31648–58. 16006557 10.1074/jbc.M504395200

[CR22] Makwana V, Ryan P, Patel B, Dukie SA, Rudrawar S. Essential role of O-GlcNAcylation in stabilization of oncogenic factors. Biochim Biophys Acta Gen Subj. 2019;1863:1302–17. 31034911 10.1016/j.bbagen.2019.04.002

[CR23] Netsirisawan P, Chaiyawat P, Chokchaichamnankit D, Lirdprapamongkol K, Srisomsap C, Svasti J, Champattanachai V. Decreasing O-GlcNAcylation affects the malignant transformation of MCF-7 cells via Hsp27 expression and its O-GlcNAc modification. Oncol Rep. 2018;40:2193–205. 30106436 10.3892/or.2018.6617

[CR24] Park JM, Kim SY, Park D, Park JS. Effect of Edaravone therapy in Korean amyotrophic lateral sclerosis (ALS) patients. Neurol Sci. 2020;41:119–23. 31471712 10.1007/s10072-019-04055-3 PMC7223963

[CR25] Peng P, Wang J, Ding N, Zhou M, Gu Z, Shi Y, Gong C, Zhao G, Deng Y. Alteration of O-GlcNAcylation affects assembly and axonal transport of neurofilament via phosphorylation. Neurosci Lett. 2019;698:97–104. 30395884 10.1016/j.neulet.2018.11.001

[CR26] Philips T, Rothstein JD. Rodent models of amyotrophic lateral sclerosis. Curr Protoc Pharmacol. 2015;69:5671–56721. 10.1002/0471141755.ph0567s69 PMC4562058 26344214

[CR27] Pinho TS, Correia SC, Perry G, Ambrósio AF, Moreira PI. Diminished O-GlcNAcylation in Alzheimer’s disease is strongly correlated with mitochondrial anomalies. Biochim Biophys Acta Mol Basis Dis. 2019;1865:2048–59. 30412792 10.1016/j.bbadis.2018.10.037

[CR28] Poore CP, Sundaram JR, Pareek TK, Fu A, Amin N, Mohamed NE, Zheng YL, Goh AXH, Lai MK, Ip NY, et al. Cdk5-mediated phosphorylation of delta-catenin regulates its localization and GluR2-mediated synaptic activity. J Neurosci. 2010;30:8457–67. 20573893 10.1523/JNEUROSCI.6062-09.2010 PMC5283092

[CR29] Rinehart J, Vázquez N, Kahle KT, Hodson CA, Ring AM, Gulcicek EE, Louvi A, Bobadilla NA, Gamba G, Lifton RP. WNK2 kinase is a novel regulator of essential neuronal cation-chloride cotransporters. J Biol Chem. 2011;286:30171–80. 21733846 10.1074/jbc.M111.222893 PMC3191056

[CR30] Rostgaard N, Jul PH, Garmer M, Volbracht C. Increasing O-GlcNAcylation attenuates Tau hyperphosphorylation and behavioral impairment in rTg4510 Tauopathy mice. J Integr Neurosci. 2023;22:135. 37735138 10.31083/j.jin2205135

[CR31] Ryan P, Xu M, Davey AK, Danon JJ, Mellick GD, Kassiou M, Rudrawar S. O-GlcNAc modification protects against protein misfolding and aggregation in neurodegenerative disease. ACS Chem Neurosci. 2019;10:2209–21. 30985105 10.1021/acschemneuro.9b00143

[CR32] Shan X, Vocadlo DJ, Krieger C. Reduced protein O-glycosylation in the nervous system of the mutant SOD1 Transgenic mouse model of amyotrophic lateral sclerosis. Neurosci Lett. 2012;516:296–301. 22521585 10.1016/j.neulet.2012.04.018

[CR33] Sihag RK, Jaffe H, Nixon RA, Rong X. Serine-23 is a major protein kinase A phosphorylation site on the amino-terminal head domain of the middle molecular mass subunit of neurofilament proteins. J Neurochem. 1997;72:491–9. 10.1046/j.1471-4159.1999.0720491.x 9930720

[CR34] Singh V, Ram M, Kumar R, Prasad R, Roy BK, Singh KK. Phosphorylation: implications in cancer. Protein J. 2017;36:1–6. 28108801 10.1007/s10930-017-9696-z

[CR35] Skorobogatko Y, Landicho A, Chalkley RJ, Kossenkov AV, Gallo G, Vosseller K. O-linked β-*N*-acetylglucosamine (O-GlcNAc) site thr-87 regulates synapsin I localization to synapses and size of the reserve pool of synaptic vesicles. J Biol Chem. 2014;289:3602–12. 24280219 10.1074/jbc.M113.512814 PMC3916560

[CR36] Song H, Ma J, Bian Z, Chen S, Zhu J, Wang J, Huang N, Yin M, Sun F, Xu M, et al. Global profiling of O-GlcNAcylated and/or phosphorylated proteins in hepatoblastoma. Signal Transduct Target Ther. 2019;4:40–9. 31637018 10.1038/s41392-019-0067-4 PMC6799812

[CR37] Taylor JP, Brown RH, Cleveland DW. Decoding ALS: from genes to mechanism. Nature. 2016;539:197–206. 27830784 10.1038/nature20413 PMC5585017

[CR38] Torres CR, Hart GW. Topography and polypeptide distribution of terminal N-acetylglucosamine residues on the surfaces of intact lymphocytes. Evidence for O-linked GlcNAc. J Biol Chem. 1984;259:3308–17. 6421821

[CR39] Uttamapinant C, Tangpeerachaikul A, Grecian S, Clarke S, Singh U, Slade P, Gee KR, Ting AY. Fast, cell-compatible click chemistry with copper-chelating Azides for biomolecular labeling. Angew Chem Int Ed. 2012;51:5852–6. 10.1002/anie.201108181 PMC3517120 22555882

[CR40] Wang AC, Jensen EH, Rexach JE, Vinters HV, Hsieh-Wilson LC. Loss of O-GlcNAc glycosylation in forebrain excitatory neurons induces neurodegeneration. Proc. Natl. Acad. Sci. U. S. A. 2016;113:15120–15125. 10.1073/pnas.1606899113 PMC5206508 27956640

[CR41] Wang S, Yang F, Petyuk VA, Shukla AK, Monroe ME, Gritsenko MA, Rodland KD, Smith RD, Qian WJ, Gong CX, et al. Quantitative proteomics identifies altered O-GlcNAcylation of structural, synaptic and memory-associated proteins in Alzheimer’s disease. J Pathol. 2017;243:78–88. 28657654 10.1002/path.4929 PMC5647145

[CR42] Wang X, Li W, Marcus J, Pearson M, Song L, Smith K, Terracina G, Lee J, Hong KLK, Lu SX, et al. MK-8719, a novel and selective O-GlcNAcase inhibitor that reduces the formation of pathological Tau and ameliorates neurodegeneration in a mouse model of tauopathy. J Pharmacol Exp Ther. 2020;374:252–63. 32493725 10.1124/jpet.120.266122

[CR43] Xu L, Liu T, Liu L, Yao X, Chen L, Fan D, Zhan S, Wang S. Global variation in prevalence and incidence of amyotrophic lateral sclerosis: a systematic review and meta-analysis. J Neurol. 2020;267:944–53. 31797084 10.1007/s00415-019-09652-y

[CR45] Yang X, Qian K. Protein O-GlcNAcylation: emerging mechanisms and functions. Nat Rev Mol Cell Biol. 2017;18:452–65. 28488703 10.1038/nrm.2017.22 PMC5667541

[CR44] Yang F, Beltran-Lobo P, Sung K, Goldrick C, Croft CL, Nishimura A, Hedges E, Mahiddine F, Troakes C, Golde TE, et al. Reactive astrocytes secrete the chaperone HSPB1 to mediate neuroprotection. Sci Adv. 2024;10:eadk9884. 38507480 10.1126/sciadv.adk9884 PMC10954207

[CR46] Yuan A, Rao MV, Veeranna Nixon RA. Neurofilaments and neurofilament proteins in health and disease. Cold Spring Harb Perspect Biol. 2017;9. 10.1101/cshperspect.a018309 PMC5378049 28373358

[CR47] Zhang L, Bai W, Peng Y, Lin Y, Tian M. Role of O-GlcNAcylation in central nervous system development and injuries: a systematic review. Mol Neurobiol. 2024;61:7075–91. 38367136 10.1007/s12035-024-04045-3

