# Supplementary material for: Characterization and chemoproteomic profiling of protein O-GlcNAcylation in SOD1-G93A mouse model
Source: Mol Med. 2025 Feb 28;31:82. doi: 10.1186/s10020-025-01134-4 (PMC11871760; doi:10.1186/s10020-025-01134-4)
Supplement: Supplementary file 3 — Supplementary Material 3 [file 10020_2025_1134_MOESM3_ESM.docx]

**Supplementary Material**

**Characterization and chemoproteomic profiling of protein O-GlcNAcylation in SOD1-G93A mouse model**

Yi Hao^1,#^, Zhongzhong Li^2,#^, Xinyan Du^2^, Qingsong Xie^1^, Dongxiao Li^3^, Shaoyuan Lei^4^, Yansu Guo^2,4,5,*^

^1^National Glycoengineering Research Center, Shandong University, Qingdao, Shandong, China.

^2^Beijing Geriatric Healthcare and Disease Prevention Center, Xuanwu Hospital, Capital Medical University, Beijing, China.

^3^Department of Neurology, The First Hospital of Hebei Medical University, Shijiazhuang, Hebei, China.

^4^Evidence-Based Medicine Center, Xuanwu Hospital, Capital Medical University, Beijing, China.

^5^Beijing Municipal Geriatric Medical Research Center, Beijing, China.

**^#^**These authors contributed equally to this work.

***Corresponding author:** Yansu Guo, Beijing Geriatric Healthcare and Disease Prevention Center, Xuanwu Hospital, Capital Medical University, Changchun Street 45, Beijing, China. Email: gys188@163.com

**Supplementary Figures**

**
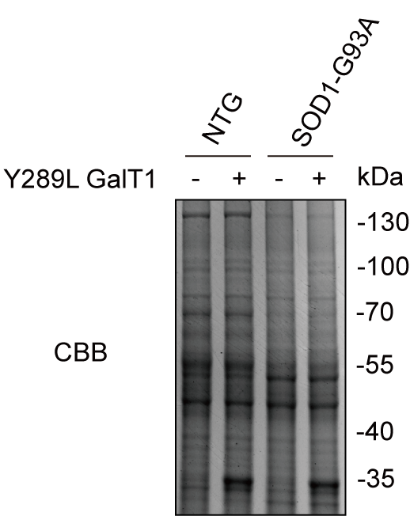
**

**Figure S1.** The uncropped gel image of Coomassie Brilliant Blue (CBB)-stained gel shown in **Figure 1**.

**
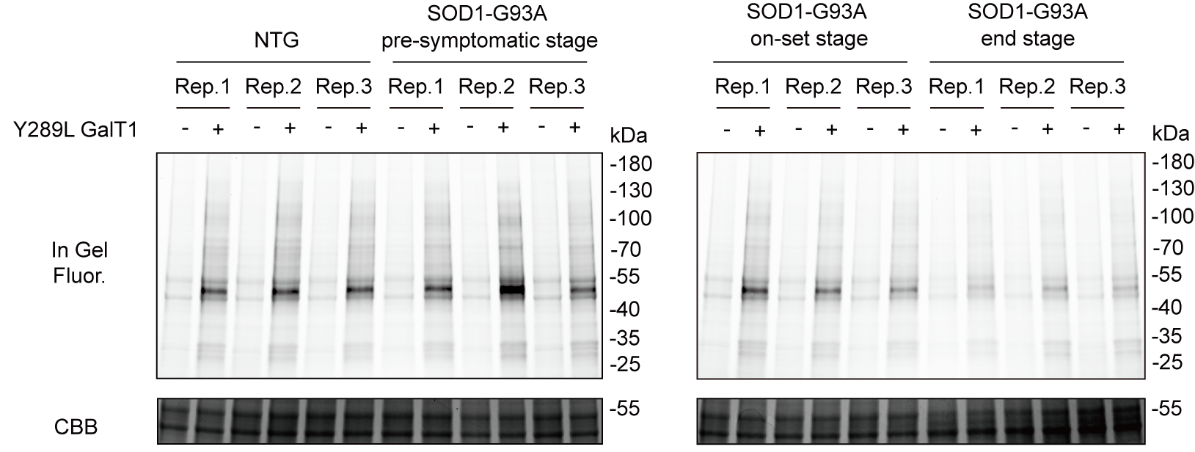
**

**Figure S2.** In-gel fluorescence scanning showing O-GlcNAc levels in lumbar spinal cord of SOD1-G93A mice at indicated disease stages. The cell lysates were incubated with UDP-GalNAz and/or Y289L GalT1, followed by conjugation with alkyne-Cy5 via click chemistry. Coomassie Brilliant Blue (CBB)-stained gels demonstrate comparable loading.

**
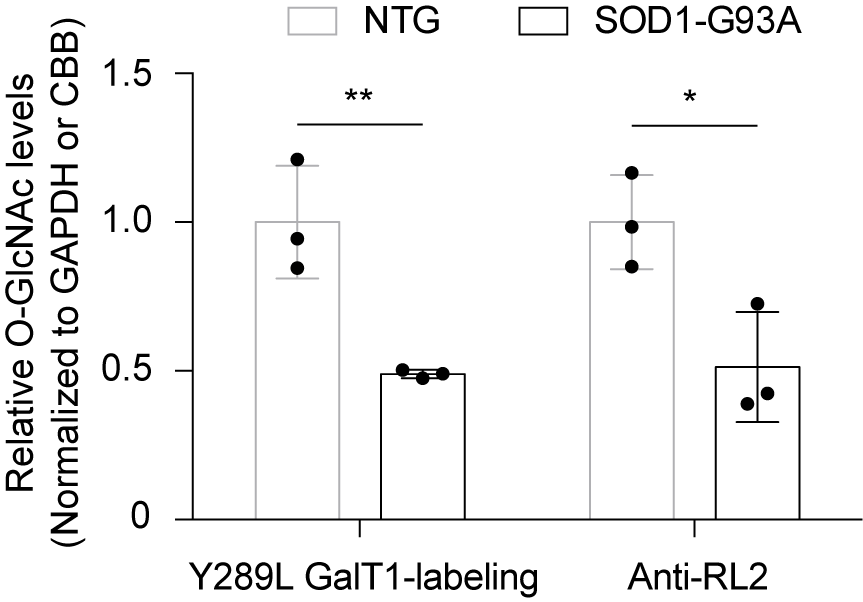
**

**Figure S3.** Bar graph showing statistical analysis of the overall O-GlcNAc level between lumbar spinal cord cells from SOD1-G93A at end stage and NTG mice. **P* < 0.05, ***P* < 0.01 (Student’s t-test).


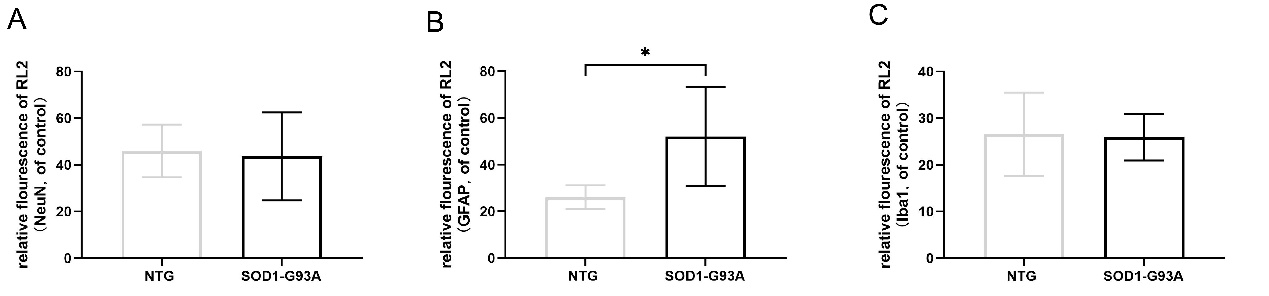


**Figure S4.** Bar graph showing quantitative analysis of O-GlcNAc-NeuN/GFAP/Iba1 colocalization in lumbar spinal cord cells from SOD1-G93A at end stage and NTG mice. **P* < 0.05, (Student’s t-test). For each group, five fluorescence images were analyzed.

**
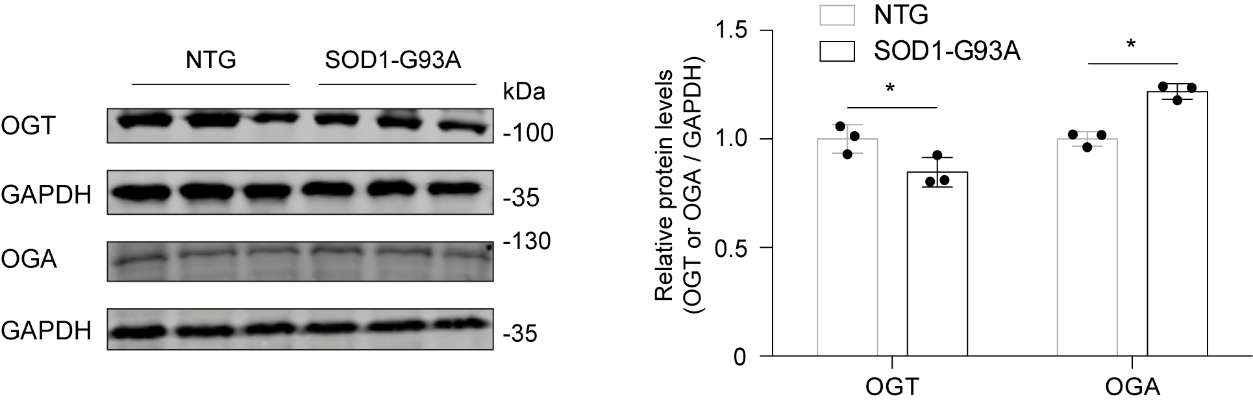
**

**Figure S5.** Immunoblots comparing the expression of OGT and OGA in lumbar spinal cord from the other three SOD1-G93A at end stage and NTG mice. Anti-GAPDH blot demonstrates comparable loading. Bar graph showing statistical analysis of the relative protein level. **P* < 0.05 (Student’s t-test).


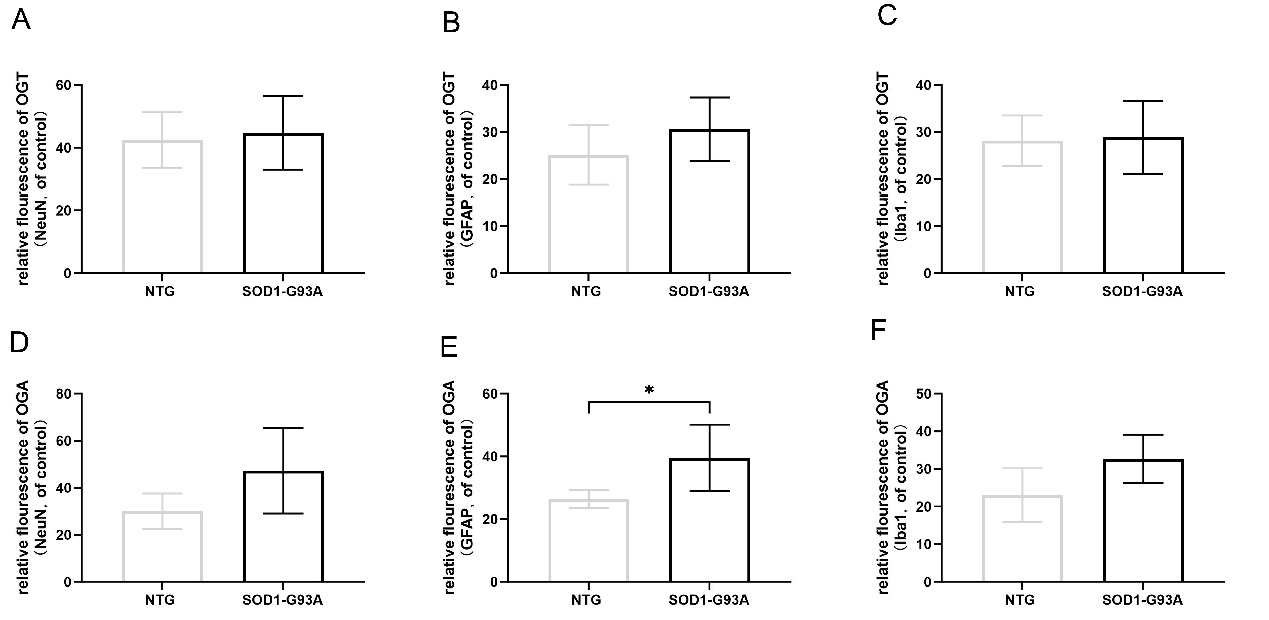


**Figure S6.** Bar graph showing quantitative analysis of OGT/OGA-NeuN/GFAP/Iba1 colocalization in lumbar spinal cord cells from SOD1-G93A at end stage and NTG mice. **P* < 0.05, (Student’s t-test). For each group, five fluorescence images were analyzed.

**
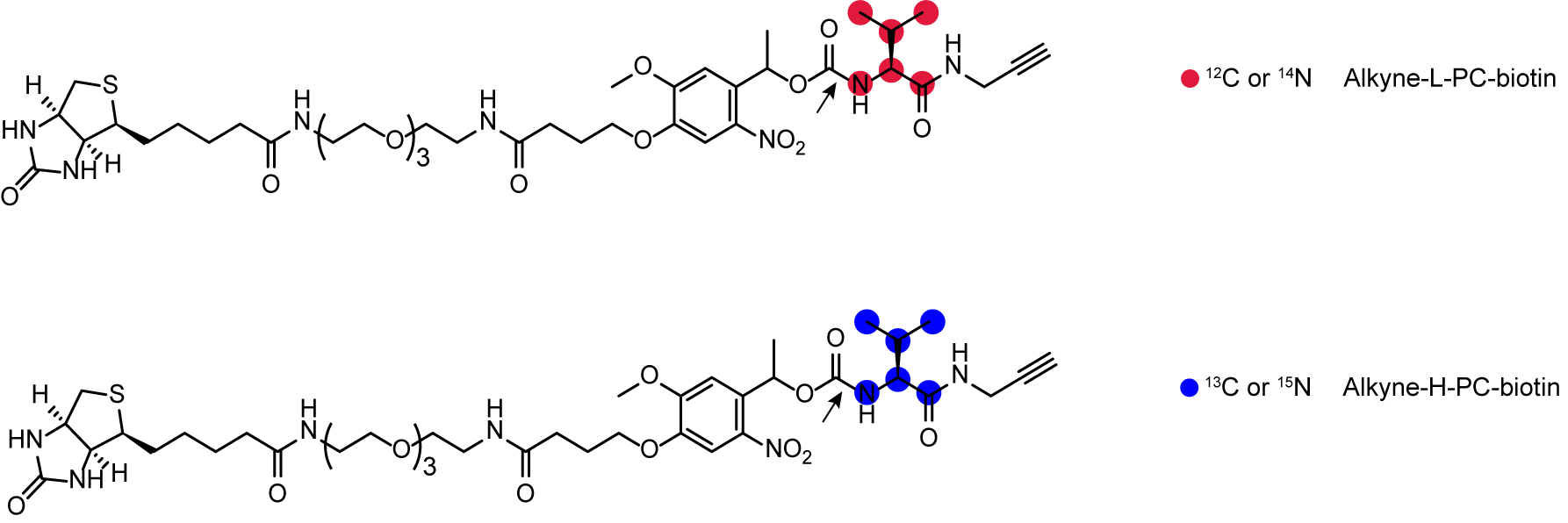
**

**Figure S7.** Chemical structures of alkyne-L/H-PC-biotin in isoPTOP (Liu et al. 2022). The arrow indicates photo-cleavage site.

**
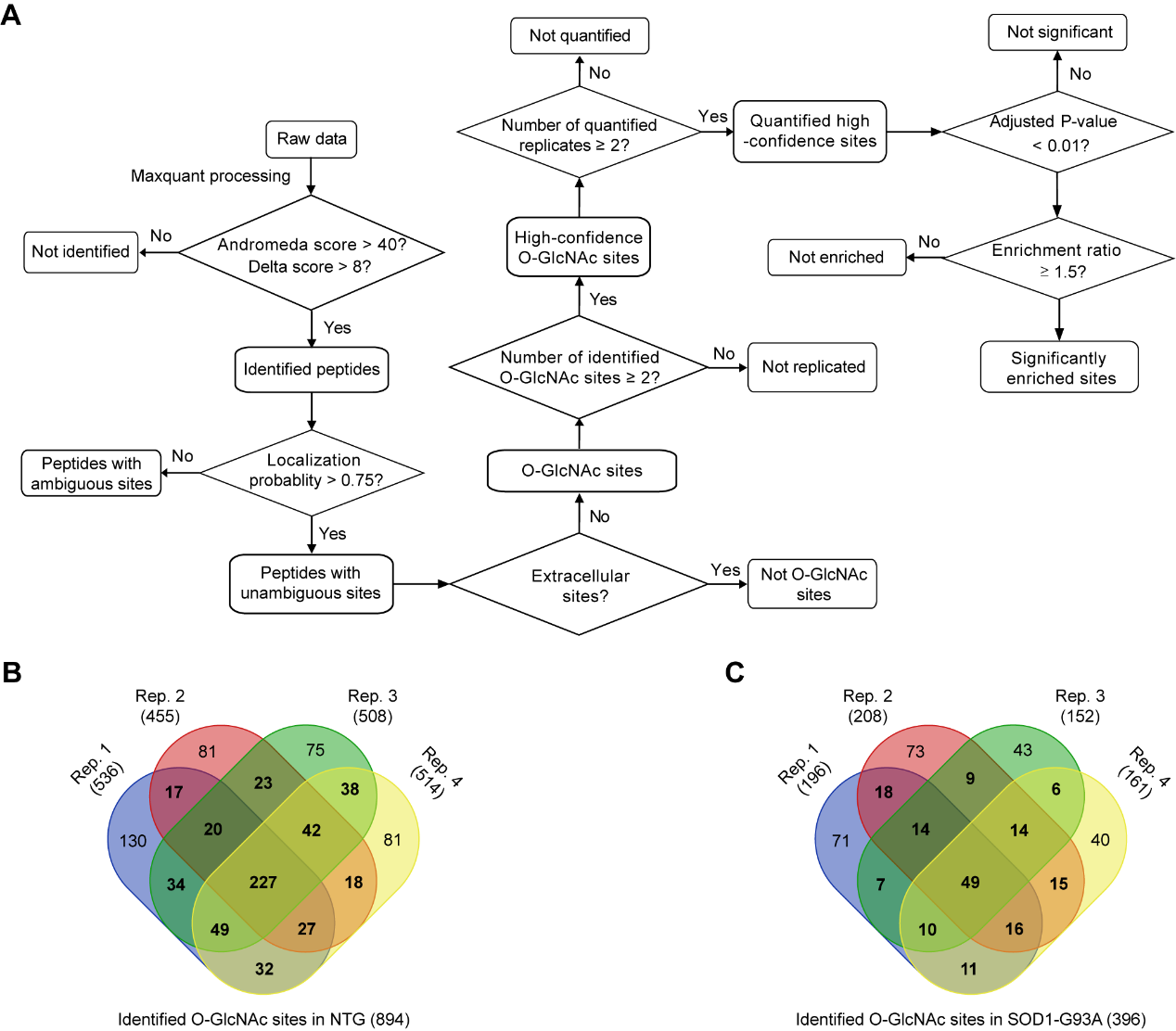
**

**Figure S8.** Proteomic identification and quantification of O-GlcNAc sites in lumbar spinal cord of NTG and end-stage SOD1-G93A mice. (**A**) Flowchart of the streamlined process for MS data analysis. (**B**), (**C**) Overlap of identified O-GlcNAc sites in four biological replicates from NTG (**B**) and SOD1-G93A (**C**) mice.

**
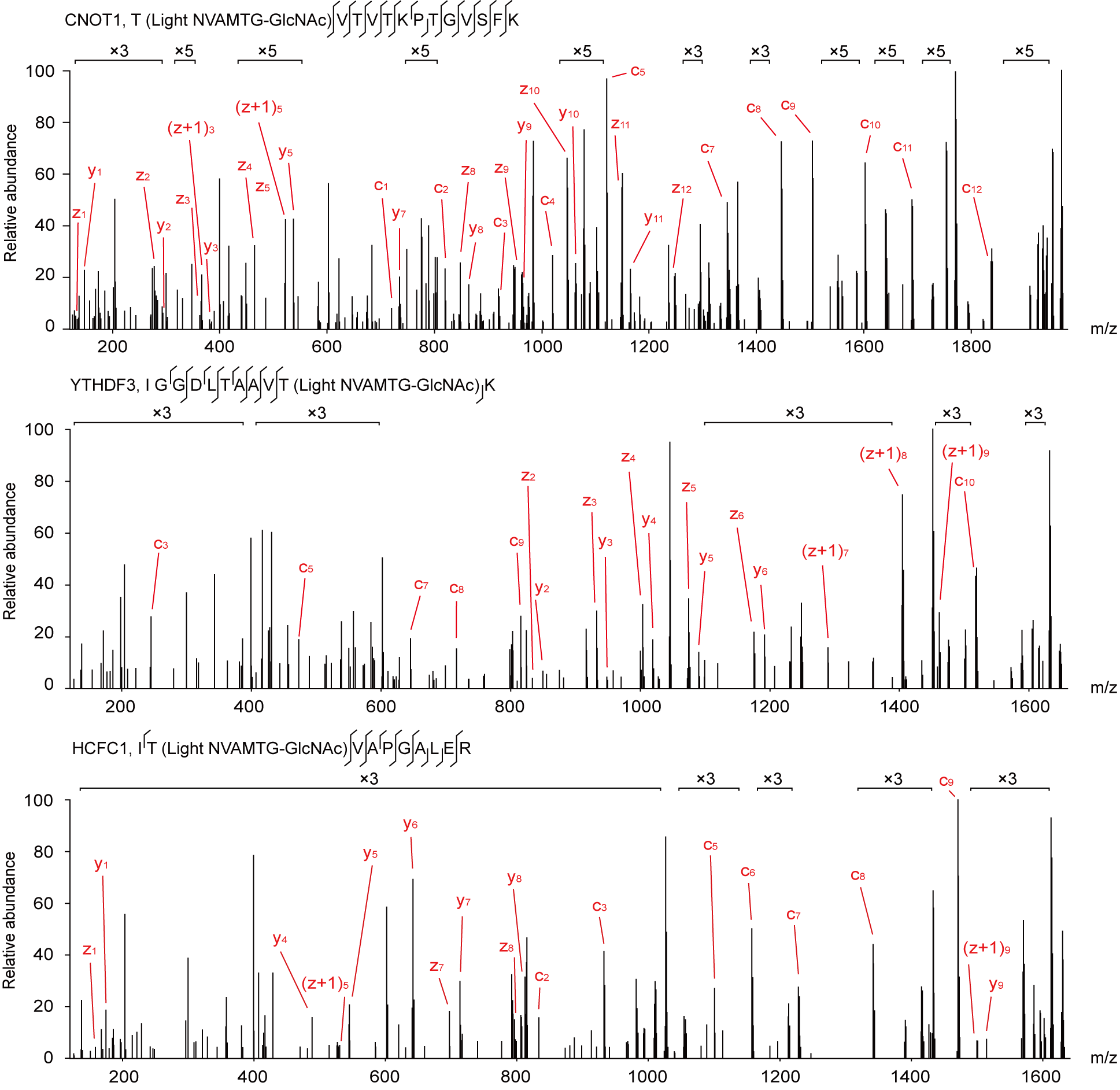
**

**Figure S9.** Representative MS/MS spectra of the identified glycopeptides containing a “light” NVAMTG-GlcNAc site by isoPTOP. NVAMTG, short for the *N*-(L-valyl-aminomethyltriazolylacetyl) galactosamine group. As reported (Liu et al. 2022), the isoPTOP strategy results in a “light” NVAMTG group on the O-GlcNAc moiety of O-GlcNAcylated peptides captured by alkyne-L-PC-biotin.

**
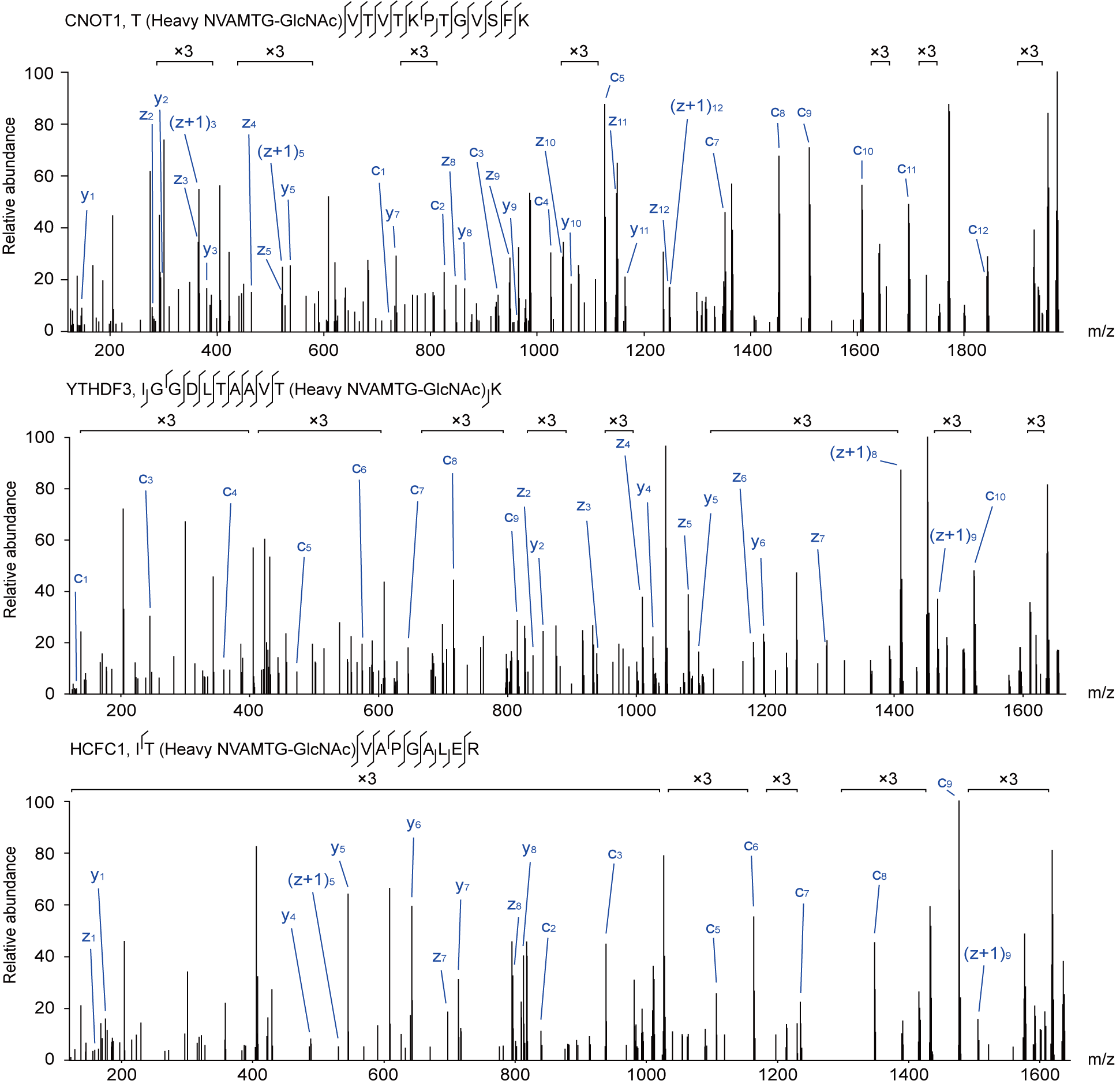
**

**Figure S10.** Representative MS/MS spectra of the identified glycopeptides containing a “heavy” NVAMTG-GlcNAc site by isoPTOP. As reported (Liu et al. 2022), the isoPTOP strategy would generate a “heavy” NVAMTG group on the O-GlcNAc moiety of O-GlcNAcylated peptides captured by alkyne-H-PC-biotin.

**Supplementary Tables**

Table S1. List of identified O-GlcNAc sites in NTG and SOD1-G93A mice. See the attached excel file.

Table S2. List of quantified O-GlcNAc sites in NTG and SOD1-G93A mice. See the attached excel file.

**Supplementary References**

Liu J, Hao Y, Wang C, Jin Y, Yang Y, Gu J, Chen X. An optimized isotopic photocleavable tagging strategy for site-specific and quantitative profiling of protein O-GlcNAcylation in colorectal cancer metastasis. ACS Chem. Biol. 2022;17:513–520.
